# Thyroid Troubles: A Case of Hypothyroidism-Associated Recurrent Massive Pleural Effusion

**DOI:** 10.7759/cureus.58072

**Published:** 2024-04-11

**Authors:** Fawwad A Ansari, Sammudeen Ibrahim, Anis Abo Baker, Mubashira Aftab, Christopher March

**Affiliations:** 1 Internal Medicine, Piedmont Athens Regional Medical Center, Athens, USA; 2 Internal Medicine, Fazaia Medical College, Islamabad, PAK

**Keywords:** endocrine disorders, myxedema, thyroid hormone, pleural effusion, hypothyroidism

## Abstract

Pleural effusions can be secondary to several different etiologies. Sometimes, they can be related to hypothyroidism. We present a case of massive pleural effusion resulting from hypothyroidism. A 75-year-old male with a history of liver cirrhosis, hypothyroidism, and medication non-adherence presented to the emergency department (ED) with shortness of breath and altered mental status. Physical exam and chest imaging were consistent with right-sided pleural effusion. Effusion was exudative. Multiple recurrences complicated the hospitalization despite thoracentesis and pleurodesis. Labs revealed hypothyroidism, and finally, the patient was started on hormone replacement, resulting in the resolution of the effusion. Pleural effusion is a rare manifestation of hypothyroidism, thought to be mediated by vascular endothelial factors. Pleural fluid analysis shows both exudative and transudative patterns. Hormonal replacement is the mainstay of treatment. Clinicians need to be aware of the rare etiologies of pleural effusion. Depending on the patient's presentation, due work-up should be done to ensure a timely diagnosis and management.

## Introduction

Hypothyroidism is a systemic endocrine disorder resulting from inadequate synthesis, secretion, or biological effects of thyroxine, leading to a myriad of nonspecific clinical presentations [1]. Pleural effusion (PEF), defined as the accumulation of fluid in the pleural space between the parietal and the visceral pleura due to an imbalance between fluid formation and removal [2,3], is an uncommon manifestation of hypothyroidism. Massive pleural effusion from hypothyroidism is exceptionally rare [4]. Some of the more common causes of PEFs include congestive heart failure, nephrotic syndrome, malignancy, and pneumonia [4]. Herein, we present a patient with hypothyroidism-induced pleural effusion, providing insight into the clinical features, diagnostic workup, management, and outcome.

## Case presentation

A 75-year-old Caucasian male with a history of liver cirrhosis, chronic kidney disease, hypothyroidism, and medication non-adherence presented to the emergency department (ED) in May 2023, with progressively worsening shortness of breath and altered mental status via the emergency medical service. Reportedly, the patient had gradually worsening shortness of breath for the past few days. He had some associated right-sided chest discomfort. On the presentation day, he was reported to be confused and altered in sensorium. He was reported to be non-verbal and minimally responsive. He had no preceding head trauma, fever, cough, focal weakness, orthopnea, paroxysmal nocturnal dyspnea, or recent long-distance travel. Family also reported that the patient had not been taking his home medication, levothyroxine, on a regular basis.

Vitals signs revealed a temperature of 91.9 F (Fahrenheit), pulse rate of 53 beats per minute, blood pressure (BP) 156/106 mmHg, and oxygen saturation (SpO2) of 89% on room air. On examination, the patient was in obvious respiratory distress, lethargic, and oriented only to person. Chest examination revealed markedly diminished breath sounds and dullness to percussion over the right lung base. The extremity exam revealed bilateral non-pitting pedal edema. The remainder of the examination was unremarkable.

Investigation (Table 1) revealed a white blood cell count (WBC) of 6.0 x 10^3^/mm3, hemoglobin of 12.9 g/dL, and creatinine of 3.25 mg/dL (baseline of 1.4-1.9). Liver function and lactate were within normal limits.

**Table 1:** Laboratory workup for the patient mm3: cubic millimeters; g/dL: grams per deciliter; mg/dL: milligrams per deciliter; IU/L: international units per liter; ALT: alanine aminotransferase; AST: aspartate aminotransferase; ALP: alkaline phosphatase

Variable	Labs (on admission)	Reference range
White blood cells (cells/mm3)	6000	4.5 to 11.0
Hemoglobin (g/dL)	12.9	12.0 to 16.0
Creatinine (mg/dl)	3.25	0.5 to 1.1
ALT (IU/L)	15	10.0 to 40.0
AST (IU/L)	37	10.0 to 40.0
ALP (IU/L)	98	30.0 to 120.0
Total bilirubin (mg/dL)	0.9	0.3 to 1.0

**Figure 1:**
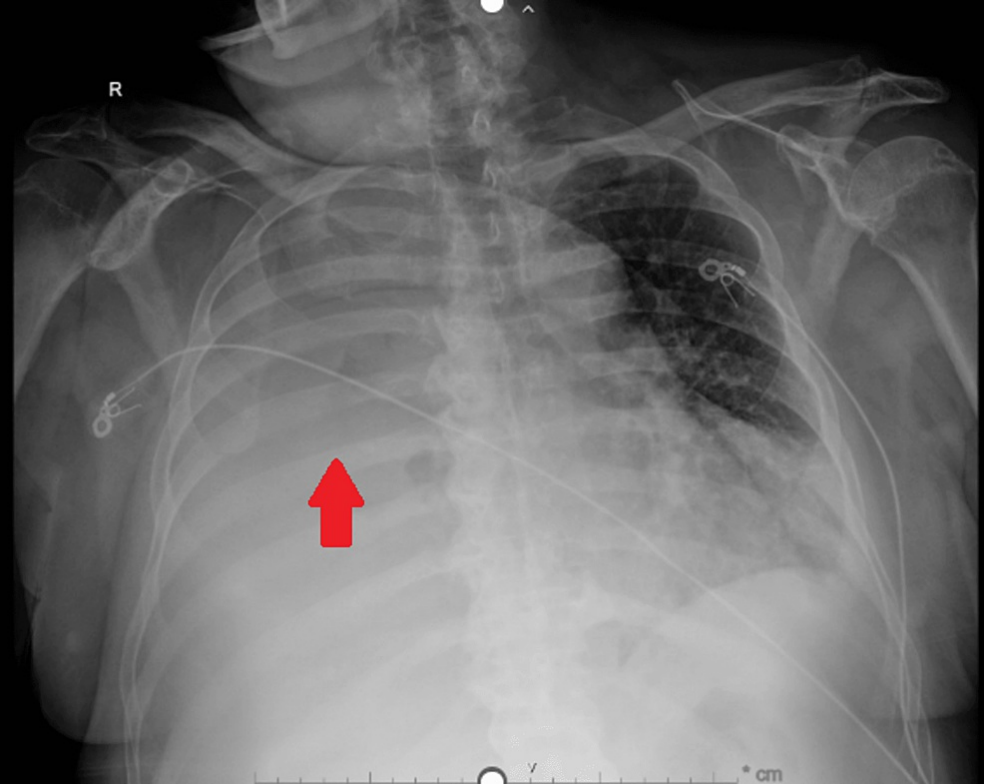
Chest radiograph Chest X-ray shows severe right-sided pleural effusion with complete opacification of right hemithorax (arrow)

**Figure 2:**
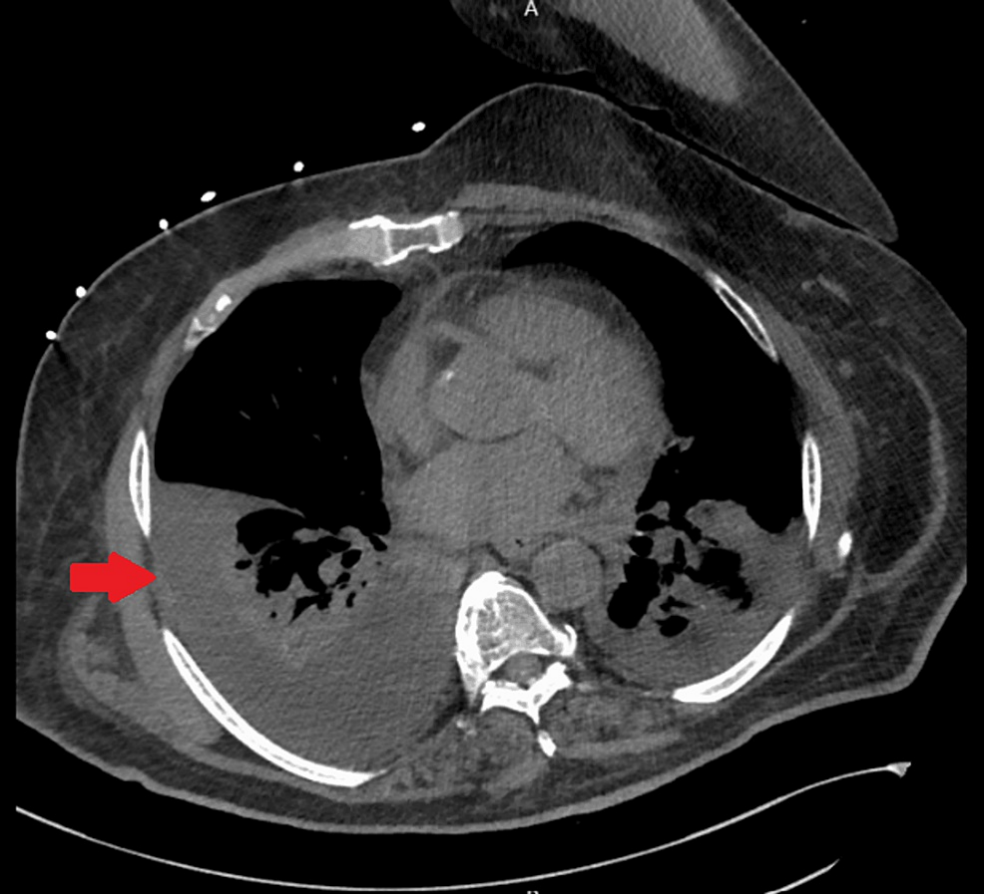
Axial view of CT-Chest without contrast CT-Chest shows a large right (arrow) and small left pleural effusion with lower lobe atelectasis

Chest X-ray demonstrated severe right-sided pleural effusion with complete opacification of right hemithorax (Figure 1), prompting further evaluation. A chest CT scan on 5/12/2023 (second day of admission) showed a large right and small left pleural effusion with lower lobe atelectasis and mild ground-glass opacities in the right upper lobe without any associated adenopathy (Figure 2).

The patient underwent thoracentesis with pleural fluid analysis showing exudative physiology, which was inconsistent with hepatic hydrothorax in the setting of known liver cirrhosis (Table 2). Other laboratory investigations were unremarkable, including pleural fluid cell count, glucose, pH, and cytology. Given the persistence of the altered mental status, thyroid function tests and serum cortisol were checked, which revealed a thyroid-stimulating hormone (TSH) of 323.5 uIU/mL; cortisol level was 13 mcg/dL. The patient's home dose of oral levothyroxine 175 mcg was continued. The hospital course was complicated by re-accumulation of exudative pleural fluid requiring multiple thoracenteses to relieve dyspnea. A chest tube was eventually placed. Having ruled out more common etiologies of exudative effusion, including infection and malignancy, severe untreated hypothyroidism was pursued as the likely etiology for pleural effusion. On day five of admission, the patient was switched to intravenous levothyroxine, given the overall patient presentation and concern for myxedema coma. He was also commenced on intravenous methylprednisolone 20 mg every eight hours for two days. The patient received intravenous levothyroxine for three days, and ultimately, levothyroxine was later changed to the oral route on day eight of treatment, as the patient's mentation had improved. Repeat TSH was 101.7 uIU/mL. Patient had a prolonged hospital course of 21 days and the patient remained on oral levothyroxine, and the patient's labs showed an improved TSH of 15 uIU/mL with free T4 of 0.90 ng/dL at the time of discharge, with significant improvement in mental status (Table 3). A repeat chest X-ray revealed the resolution of pleural effusion. The patient was discharged on oral levothyroxine 175 mcg daily with outpatient follow-up. Patient had a follow-up in July 2023, almost a month after discharge. His repeat TSH was 3.94 uIU/mL with free T4 of 1.84 (mildly elevated). Chest X-ray showed low lung volumes with possible mild right basilar atelectasis without any appreciable pleural effusion. Same dose of levothyroxine was continued.

**Table 2:** Pleural fluid analysis LDH: lactate dehydrogenase

Fluid Analysis	5/10/2023	5/12/2023 (2nd day)	5/30/2023
Pleural fluid LDH/serum LDH	146/279 (> 2/3 ULN of S. LDH)	144/227 (> 0.6)	507/147 (> 0.6)
Pleural fluid protein/serum protein	4.9/6.8 (>0.5)	4.5/5.7 (>0.5)	3.9/6.2 (>0.5)

**Table 3:** Thyroid function test uIU/mL: micro International Unit/milliliter; ng/dL: nanogram/deciliter; TSH: thyroid-stimulating hormone; FT4: free T4

Thyroid Function Test	5/10/2023 (admission)	5/18/2023 (8th day)	6/1/2023	6/28/2023 (Follow-up)
TSH (uIU/ml)	323.6	101.7	15.3	3.94
FT4 (ng/dL)	0.29	0.49	0.90	1.84

## Discussion

Hypothyroidism is a very ubiquitous disease with nonspecific clinical manifestations, including easy fatigability, dry and coarse skin, cold extremities, decreased appetite, hoarse voice, weight gain, depression, and psychomotor retardation [1]. Mild serous effusions such as ascites, pericardial, and pleural effusions are not uncommonly seen but are typically asymptomatic [5]. In rare cases, massive pleural effusion presenting with respiratory compromise may be the primary presentation. Owing to this nonspecific presentation, diagnosis is usually biochemical and is characterized by elevated serum TSH levels. 

The incidence of PEF in hypothyroidism has been estimated at 10 to 30%, associated with small fluid accumulations and of limited clinical significance [6]. However, this may be overestimated as most of the pleural effusion in hypothyroidism is related to comorbid conditions [7]. The exact incidence of hypothyroidism-induced PEF remains unclear [7]. Massive PEF has been reported as an exceedingly rare yet severe complication of decompensated hypothyroidism, which is underrecognized [7]. This is thought to be due to longstanding untreated hypothyroidism [6]. Studies have reported a mortality rate of approximately 60%, making an early diagnosis crucial for a favorable clinical outcome [8]. 

The pathophysiology of hypothyroidism-induced PEF has been postulated to be due to increased systemic capillary membrane permeability mediated by vascular endothelial growth factor (VEGF) and disruption in electrolyte metabolism related to hypothyroidism [4,8]. The resultant extravasation of albumin and disturbances in lymphatic drainage led to exudative physiology being commonly demonstrated on pleural fluid analysis in cases of hypothyroidism-induced PEFs [4]. Although initially thought to be related to autoimmune-related serositis seen commonly in concomitant autoimmune disorders associated with Hashimoto's thyroiditis, this hypothesis does not explain the occurrence of pleural effusion in iodine deficiency-related hypothyroidism. Significantly, the effusions can sometimes be borderline between exudative and transudative, with no evidence of inflammation [7]. There have also been reports of unilateral and bilateral cases of hypothyroidism-induced pleural effusion [8]. Ultimately, these variations pose a challenge to the diagnosis of hypothyroidism-related pleural effusion. 

In our patient, we initially made a diagnosis of pleural effusion with differentials of parapneumonic effusion, tuberculosis (TB), hepatic hydrothorax, heart failure, and malignant effusion. Fluid analysis was exudative, making heart failure and hepatic hydrothorax less likely, especially given that the patient had evidence of well-compensated liver function. There was concern for parapneumonic effusion from possible aspiration, given the patient had altered mental status, but the fluid analysis revealed no evidence of infection. Fluid cytology was also negative for malignant cells. However, due to the test's low sensitivity, we repeated thoracentesis, but cytology results remained negative [8]. Considering the patient had persistent altered mental status with psychomotor retardation and bilateral non-pitting pedal edema, the TSH was checked, which was markedly elevated with low free T4 and T3 levels. A review of the literature revealed rare cases of pleural effusion in hypothyroidism, which were treated with timely hormonal replacement therapy [9]. Our patient had already been on oral levothyroxine since admission. We switched him to intravenous treatment considering suspicion of possible myxedema coma contributing to altered mentation and pleural effusion. The American Thyroid Association recommends an intravenous levothyroxine dose of 75% of the oral dose. Intravenous injection is firmly not recommended for long-term treatment due to its rapid and significant effect on thyroid hormone homeostasis [10]. Hence, intravenous treatment was pursued for three days only. The patient was monitored closely, and his thyroid function improved on the therapy. His mentation also improved, and no further pleural effusions were noted. The patient was later switched back to oral levothyroxine therapy. 

Although pleural effusion is commonly seen in hypothyroidism, it is rarely caused by hypothyroidism. Even more rarely do they become clinically significant, thus posing diagnostic challenges. Nonetheless, other competing diagnoses must be ruled out first, including infectious, neoplastic, autoimmune, and metabolic diseases. Given the complete resolution of the pleural effusion after hormonal replacement with no recurrence after achieving a euthyroid state, it suggests that the pleural effusion was likely related to hypothyroidism. 

## Conclusions

This case report highlights the association between hypothyroidism and massive pleural effusion, emphasizing the importance of considering thyroid dysfunction in patients presenting with unexplained pleural effusions. Early diagnosis and treatment of hypothyroidism are essential to prevent further complications and improve patient outcomes. Additional research is warranted to elucidate this association's exact mechanisms and identify potential therapeutic interventions. 

## References

[1] Yuan GQ, Yan QL, He MH (2020). A case of pleural effusion caused by hypothyroidism. J Biosci Med.

[2] Saguil A, Wyrick K, Hallgren J (2014). Diagnostic approach to pleural effusion. Am Fam Physician.

[3] Karkhanis VS, Joshi JM (2012). Pleural effusion: diagnosis, treatment, and management. Open Access Emerg Med.

[4] Lee JH, Park M, Park MJ, Jo YS (2018). Massive pleural and pericardial effusion due to hypothyroidism in a patient with a surgically treated thyroid-stimulating hormone-producing pituitary adenoma. Acta Clin Belg.

[5] Guha B, Krishnaswamy G, Peiris A (2002). The diagnosis and management of hypothyroidism. South Med J.

[6] Gomes Santos P, Calças Marques R, Martins Dos Santos P, Carreira da Costa C, Mogildea M (2023). Ascites, pleural, and pericardial effusion in primary hypothyroidism: a rare case report. Cureus.

[7] Rehan M, Alam MT, Imran K, Farrukh SZ, Masroor M, Kumar P (2013). The frequency of various diseases in patients presenting with pleural effusion. Gomal J Med Sci.

[8] Gottehrer A, Roa J, Stanford GG, Chernow B, Sahn SA (1990). Hypothyroidism and pleural effusions. Chest.

[9] Pairman L, Beckert LE, Dagger M, Maze MJ (2022). Evaluation of pleural fluid cytology for the diagnosis of malignant pleural effusion: a retrospective cohort study. Intern Med J.

[10] Liu H, Li W, Zhang W, Sun S, Chen C (2023). Levothyroxine: conventional and novel drug delivery formulations. Endocr Rev.

